# Lasing in strained germanium microbridges

**DOI:** 10.1038/s41467-019-10655-6

**Published:** 2019-06-20

**Authors:** F. T. Armand Pilon, A. Lyasota, Y.-M. Niquet, V. Reboud, V. Calvo, N. Pauc, J. Widiez, C. Bonzon, J. M. Hartmann, A. Chelnokov, J. Faist, H. Sigg

**Affiliations:** 1 Laboratory for Micro- and Nanotechnology, Paul Scherrer Institut, 5232 Villigen, Switzerland; 2 Univ. Grenoble Alpes, CEA, LETI, 38054 Grenoble, France; 3 Univ. Grenoble Alpes, CEA, IRIG-DePhy, 38054 Grenoble, France; 4 Institute for Quantum Electronics, ETH Zürich, 8093 Zürich, Switzerland

**Keywords:** Mid-infrared photonics, Optoelectronic devices and components, Semiconductor lasers

## Abstract

Germanium has long been regarded as a promising laser material for silicon based opto-electronics. It is CMOS-compatible and has a favourable band structure, which can be tuned by strain or alloying with Sn to become direct, as it was found to be required for interband semiconductor lasers. Here, we report lasing in the mid-infrared region (from *λ* = 3.20 μm up to *λ* = 3.66 μm) in tensile strained Ge microbridges uniaxially loaded above 5.4% up to 5.9% upon optical pumping, with a differential quantum efficiency close to 100% with a lower bound of 50% and a maximal operating temperature of 100 K. We also demonstrate the effect of a non-equilibrium electron distribution in **k**-space which reveals the importance of directness for lasing. With these achievements the strained Ge approach is shown to compare well to GeSn, in particular in terms of efficiency.

## Abstract (web summary)

Germanium (based) lasers are a promising route towards a fully CMOS-compatible light source, key to the further development of silicon photonics. Here, the authors realize lasing from strained germanium microbridges up to 100 K, finding a quantum efficiency close to 100%.

## Introduction

Si photonics is seen as a major enabler for optical data communication, signal processing and sensing applications. The fabrication of photonic circuits relies on processes and protocols similar to the commonly implemented complementary metal-oxide-semiconductor (CMOS) technology, and thus it is restricted to a well-defined set of materials, preferentially from the group IV atomic column, like silicon. Many of the required photonic components have been successfully developed in such CMOS-like technology^1–4^, but the key element, an electrically driven laser source fully integrated on Si, is still missing. Despite the constant progress of heterogeneous integration of III-V based lasers on Si^5–8^, the development of optical sources realized in group IV still represents a long term interest in the Si-photonics field. Germanium, thanks to its CMOS compatibility and near direct bandgap configuration—140 meV offset between the conduction band minima at Γ and L—is thus the best candidate, providing that the directness can be improved. Efforts in this direction follow three approaches: strong doping with donors^9^, alloying with Sn^10–13^ and the application of tensile strain^14–17^.

While alloying with Sn is now a well-established technique to achieve laser action in group IV materials^11–13^, heavy n-doping, combined with moderate tensile strain, was proposed initially as a way to achieve optical gain and lasing. Indeed, since 2010 three refereed publications report laser operation upon optical and electrical pumping^9,18,19^. These results, however, are in contradiction with spectroscopic gain measurements performed in similar materials, which showed that the accumulation of holes and the associated intervalence band absorption prevent net material gain^20,21^. The third approach, tensile strain, is very attractive as with a single parameter the band structure can be adjusted from indirect to direct. The two most common approaches include either stressor layers^16,17^ or strain amplification^22^. To date, strain levels of about 2% in the <110> biaxial configuration^16,17,23^ and more than 5% in the <100> uniaxial configuration^24–26^ are reported. Most of the theoretical models suggest that these strain values should be high enough to reach a direct bandgap, enabling the possibility of lasing action, if optical feedback is provided. Yet, both for GeSn and tensile strained Ge, the values for the offset (Δ*E*) between the Γ and L band minima, the electron and hole effective masses and the fundamental processes, such as the dynamics of scattering between valleys or Auger recombination^27^, are not well understood, making lasing gain and parasitic loss prediction unreliable. On the other hand, the fabrication of strain compatible optical cavities was demonstrated for microdiscs^16,17^ and also microbridges^28,29^, providing the experimental access to the above-mentioned quantities and to investigate the lasing^30,31^.

The purpose of this paper is to study in detail a laser based on strained undoped germanium. We report for strained Ge key lasing attributes such as an absolute power measurement demonstrating a high differential quantum efficiency when the laser operates above a sharp laser threshold as well as the onset of mode competition and linewidth narrowing. Furthermore, we discuss the temperature dependence of the lasing and present model calculations of the valley scattering from Γ to L. We argue that—for our case—lasing in Ge under pulsed excitation is helped by a non-equilibrium carrier distribution in **k**-space when the two valleys become closely aligned energetically. This effect reveals the importance of directness for lasing and—in particular—for the lasing at higher operation temperature in any of the above-mentioned configurations.

## Results

### Strained germanium microbridges

**Fig. 1:**
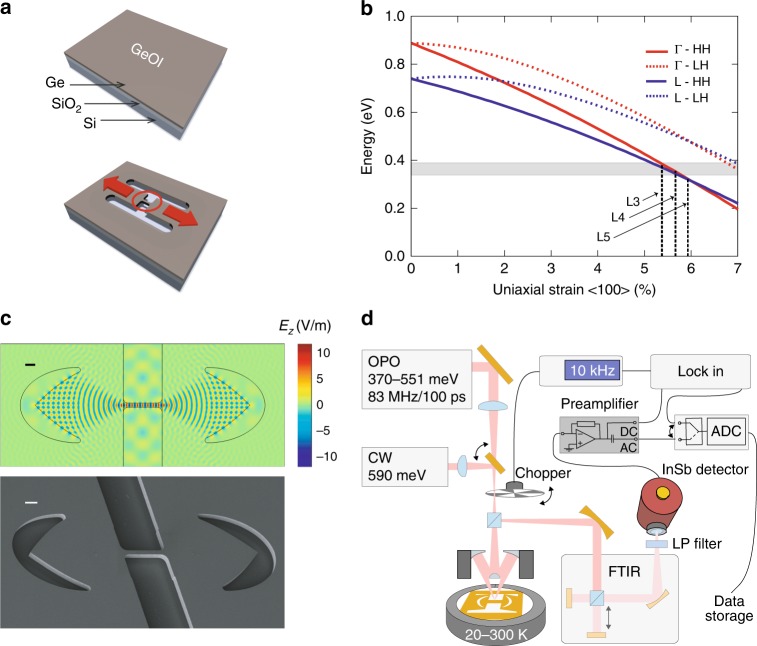
Strain engineered microbridge with optical cavity and experimental set-up. **a** Schematic illustration of a germanium-on-insulator (GeOI) substrate made of 1 µm of biaxially strained (0.16%) Ge and 1 µm of SiO_2_ on a Si (001) substrate, followed by the main processing steps. The geometry of the microbridge structure is defined by e-beam lithography and it is transferred to the Ge layer via inductively coupled plasma dry etching. The HF vapour selectively under etches the oxide, allowing the pads to relax their strain along the directions indicated by the red arrows, stretching the microbridge indicated by the red circle. **b** Evolution of the indirect, i.e. the energy transition from the heavy hole (HH) and light hole (LH) bands to L, and direct bandgap (HH and LH to Γ) in function of the uniaxial strain in the crystallographic direction <100>, calculated with the tight-binding method at 20 K, showing the crossing from the indirect to the direct configuration at about 6% of strain. The strain of the probed microbridge configurations L3, L4 and L5 at 20 K, namely 5.4, 5.7 and 5.9%, respectively, are indicated together with the energy range of the lasing emission, in grey. **c** Finite element method simulation with COMSOL of a TE cavity mode (scale bar, 2 μm) and the corresponding tilted view scanning electron microscopy (SEM) image (scale bar, 2 μm). **d** Schematic of the experimental set-up, highlighting the main elements: OPO optical parametric oscillator, CW continuous wave diode laser, FTIR Fourier-transform infrared spectrometer, LP, low pass filter and detector

For this study, we investigated suspended Ge microbridges integrated into a strain preserving optical cavity with, at low temperature, uniaxially loaded tensile strain up to 5.9%. The strain is achieved by the relaxation of two biaxially pre-strained pads^14,22^, as shown in Fig. 1a and discussed in Methods, below. Samples notations of L3, L4 and L5 refer to structures with total pad lengths of 240, 260 and 280 µm, respectively. The corresponding strain values at 20 K (5.4, 5.7 and 5.9%) follow from the pad geometry and the thermal expansions. The values of strain are obtained through temperature dependent X-ray and optical Raman scattering analysis, as explained in the Supplementary Note 6. The thus deduced strain values are incorporated in Fig. 1b, which shows the strain dependence of direct and indirect bandgaps at 20 K obtained from our tight-binding (TB) model, which allows us to calculate the full strain dependent band structure in the reciprocal space. The above-mentioned TB model, detailed in ref. ^32^ and applied in ref. ^33^, predicts an indirect to direct bandgap crossover for an uniaxial strain close to *ε*~6% at 20 K, as reported in the Supplementary Note 9. The crossover value is larger than the pseudo-potential calculation of ref. ^34^ with a crossover at *ε*~5% or the deformation potential model^35^, which anticipates the crossing at 4.4%. For comparison, the here employed tight-binding model predicts for the biaxial strain a crossing to the direct bandgap configuration around 2.1% of strain at 20 K.

Figure 1c shows the cavity mode nearfield pattern, calculated using the COMSOL MP solver, which builds in a cavity as the one shown in the SEM image, highlighting the unique design of the corner-cube mirrors here employed, whose concept is delivered in the Supplementary Note 8. For an empty cavity—i.e. when zero propagation loss is assumed—quality factors (*Q*) between 1000 to 2500 are obtained, depending on the cavity length, which is 44 (28) µm for the long (short) cavities. Throughout the paper, all the results are obtained from long cavity samples, if not explicitly stated otherwise. For long cavities, the photon round trip time is close to 1 ps and the photon decay time (decrease of energy of a factor 1/e) is ~5 ps (*Q* = 2500). Hence, to compensate for scattering losses, the amount of modal gain required before lasing can occur is at least about 140 cm^−1^, when no other losses are involved. This substantial gain is required by the only 20% filling factor of the cavity with gain material, namely the 8 µm long bridge.

The microbridge structures were excited with two kinds of pump laser; a diode laser running in continuous wave (CW) and an optical parametric oscillator (OPO) operated in pulsed mode (pulse width and repetition rate of 100 ps and 83 MHz, respectively). Both lasers run at energies below the bandgap of unstrained Ge, making the pad regions transparent for the excitations. The samples were mounted in a cryostat with a base temperature of 20 K, see Fig. 1d. Throughout the paper, the excitation intensities are defined as the time averaged power measured through a 10 µm diameter pinhole, placed at the sample position. The emission intensity is given as the averaged power harvested within the NA of the microscope. The applied calibration procedure is described in the Supplementary Note 8.

### Lasing characteristics and quantum efficiency

**Fig. 2:**
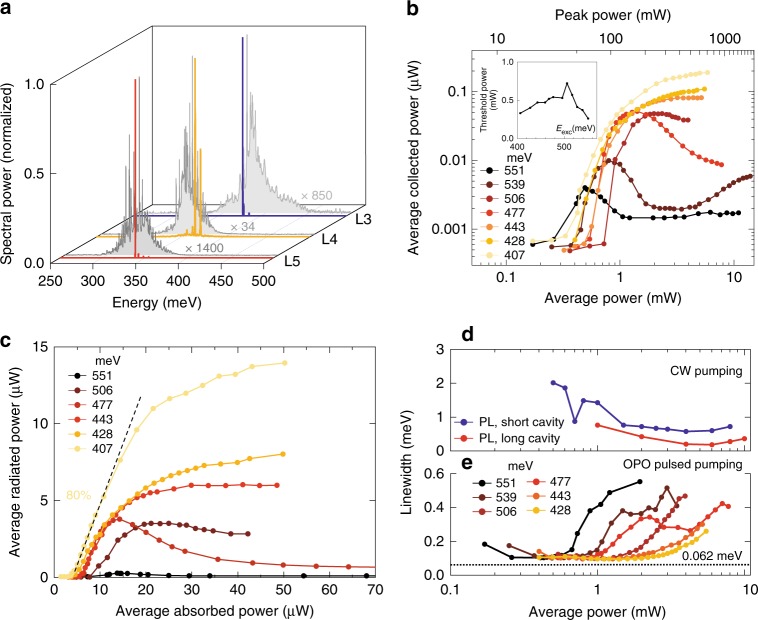
Strained Ge laser at 20 K. **a** Normalized spectra taken at 20 K of L3, L4 and L5, whose emission red-shifts with the strain. In grey colours are the photoluminescence (PL) spectra taken at about 4 mW excitation power, excited with a continuous wave (CW) diode laser operating at 590 meV energy. In blue, orange and red the corresponding lasing spectra obtained when the excitation is switched to the pulsed regime, obtained from the tuneable OPO, at an average excitation power of 2 mW, with energy of 551 meV and 428 meV, for L4 and L3, L5 samples, respectively. Per each sample, the relative magnification of the PL spectrum with respect to the corresponding laser spectrum is reported. **b** Average power collected by the objective with a numerical aperture (NA) of 0.4, as a function of the average excitation power (integrated over an area of 100 π/4 µm^2^) in pulse regime, for the L5 sample at 20 K. Curves for different excitation energies are shown. Inset: threshold dependency on the excitation energy. **c** Comparison between the average radiated power and absorbed power, for different excitation energies, for the case of the L5 sample at 20 K obtained by a conversion from **b** as explained in the main text. The value of the differential efficiency, commonly referred to as slope efficiency, is indicated for the pump energy of 407 meV. **d** Comparison of the cavity modes linewidth power dependence between two L5 samples, with short and long cavity, under CW excitation. **e** Linewidth power dependence of the main L5 lasing mode for different excitation energies in pulsed regime. Note that the reported linewidths are apodized and approaching the instrumental resolution of 0.062 meV, as indicated by the dashed line

In Fig. 2a, in grey scale, we show photoluminescence (PL) spectra taken at 20 K with an excitation energy of *E*_exc_ = 590 meV in steady state for the three differently strained bridges L3, L4 and L5. The strong periodic modulation of the intensity is attributed to the cavity modes evolving when the medium is driven to transparency by optical pumping. Superimposed in colours, we show the spectra obtained when the bridges are excited with the OPO at similar average intensities, namely 2 mW and *E*_exc_ = 551 meV and 428 meV, for L4 and L3, L5, respectively. A magnified view of the spectra of L3 and L5 is given in the Supplementary Note 4. We obtain a strong emission from just few cavity modes revealing mode competition, whose appearance we attribute to stimulated emission and optical feedback and we consider it as one of the strongest evidences of lasing. This feature is missing in the steady state regime, where the emission pattern is largely independent on the excitation intensity and can be well explained by (amplified) spontaneous emission.

The time averaged and spectrally integrated emission intensity—the so-called light-in light-out (LL) curve—for a series of excitation energies for the L5 bridge is shown in Fig. 2b on a double logarithmic plot. Beyond threshold, a strong intensity increase is observed, which rolls-over at an excitation strength that increases when decreasing the excitation energy from 551 meV to ~477 meV. By further decreasing the excitation energy, the emission intensity raises continuously up to the highest applied powers, but sub linearly with the excitation power. Further visual proof of laser action is demonstrated by an animation of the laser spectra displayed for increasing pump power (Supplementary Movie 1 and 2).

The analysis of the pump photon energy dependence of the laser threshold power is shown in the inset of Fig. 2b. Overall, the threshold power is lowest (0.26 mW) for a pumping energy of *E*_exc_ = 551 meV, it peaks at 506 meV (0.72 mW), and then it decreases again down to 0.33 mW, for *E*_exc_ = 407 meV. We attribute the presence of the local maximum threshold pumping power at *E*_exc_ = 506 meV, to a simultaneous occurrence of the intervalence band resonance between the heavy hole (HH) and light hole (LH) bands, i.e. when the energy difference between the absorbed and emitted photon, *hν*_in_–*hν*_out_, is equal to the energy difference between the HH and LH, *E*(HH)-*E*(LH). This resonance is also responsible for the presence of a Raman scattering gain that compete with inversion gain in this pump energy range^36^. This behaviour will be discussed more in detail elsewhere.

The carrier concentration at threshold (*n*_th_) is extracted from the integrated number of photons/pulse absorbed by the structure, which is calculated from the incident excitation power, weighted by the geometrical overlap (0.1) of the 10 μm diameter pump beam with the 1 µm wide and 8 µm long bridge, and the absorbed fraction, which includes Fresnel losses and multiple passes absorption. For the excitation power of 0.26 mW at excitation energy of 551 meV, our analysis yields *n*_th_ = 1.2 × 10^17^ cm^−3^, which corresponds to an overall quantum pumping efficiency of *η* = 2.8%. In order to avoid a numerical analyses of finite size effects, which are particularly important for the low energy excitations, the absorbed fraction for these energies is obtained from the experimental slope of the mode shifts^37^ normalized to the absorbed fraction deduced for the 551 meV excitation. (More details are delivered in the Supplementary Note 1). For the lowest excitation energy (*E*_exc_ = 407 meV), we thus find a threshold density of *n*_th_ = 0.6 × 10^17^ cm^−3^, which is lower by a factor of 2 with respect to the value obtained for *E*_exc_ = 551 meV, despite the higher threshold power (0.33 mW). This reflects the lower absorption of Ge at this energy and it translates to an overall quantum pump efficiency of *η* = 0.86%. Interestingly, parasitic intervalence band absorption—which is the main challenge for the doped Ge approach^20^—is at such low densities and the low temperatures strongly suppressed.

We also analysed the differential efficiency of an L5 sample, by converting the average excitation and the collected power of the light-in light-out curves of Fig. 2b to the average absorbed and radiated power of Fig. 2c, respectively. For the first conversion we use the previously calculated pumping efficiency, while for the latter one we evoke 3D calculations, computed by the RSOFT Suite (Supplementary Note 8). We obtain that about 15% of the total optical losses escapes upwards through the surface, while the remaining optical loss escapes downwards or propagates in plane, as shown in the 2D simulation plot of Fig. 1c. By mapping the Cassegrain geometry of our collecting optics to the computed far-field of the corner-cube structure (see Supplementary Fig. 8b) we computed that 9.1% of the radiation emitted out of plane by the bridge is collected by our optic bringing the total collection efficiency of the laser mode to 1.4%. The latter, together with the pumping efficiency factor, enables us to plot the inferred light-in light out of the structure, reported in Fig. 2c. At 20 K and pump energy of 407 meV, we obtain a differential efficiency of 80%, which, by converting it into the number of photons absorbed and radiated, brings the differential quantum efficiency close to 100%. This high number, however, comes with a rather large uncertainty which we estimate to be about a factor of 2 as derives from the uncertainty of the set-up calibration, the alignment of the sample and the consequent error in the far-field collection estimation as well as the neglect of other scattering channels such as surface roughness in the 3D model. By these considerations, the lower bound of our estimation is about 50%. Nevertheless, this high efficiency, when compared to the reported quantum efficiency for GeSn of about 1.5%^11^, supports our assumption of low parasitic loss and indicates strained germanium as a favourable material for efficient lasing.

The spectral linewidth of the emitted spectrum as a function of pump power are compared for both continuous wave and pulsed powers in Fig. 2d, e, respectively. The linewidth measure in continuous wave is seen to decrease continuously as a function of pump intensity, reaching 0.190 meV for the long cavity device. However, the lack of clear threshold behaviour demonstrates that these devices do not operate in the true laser regime, but are simply exhibiting emission from high *Q* modes in an active region, which becomes increasingly transparent. In fact, the linewidth observed (*Q* = 1900) is close to the one calculated for the empty cavity (*Q* = 2500). In contrast, the same devices operating in pulse operation exhibit a much narrower linewidth, down to 0.095 meV, over a large pumping power range until transient chirp increases the linewidth at highest pump intensities. Detailed spectra are given in the Supplementary Note 2. The narrowness of the lines, despite the transient nature of the excitation, indicates a well-developed laser operation. Unfortunately, in pulsed excitation regime, the spectra could not be measured far below threshold because of the low duty cycle of the pumping and hence the lack of signal.

### Temperature dependence

**Fig. 3:**
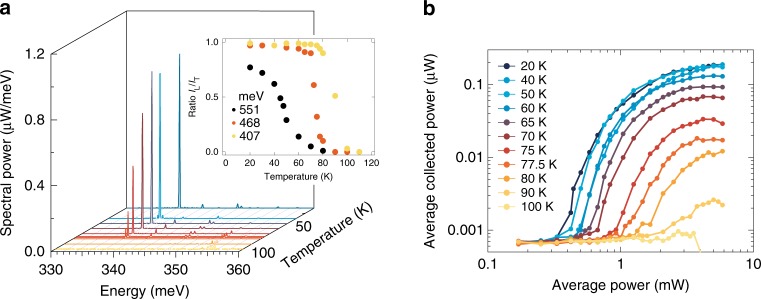
Temperature dependence of the strained Ge laser. **a** Lasing spectra of a L5 microbridge between 20 and 100 K, at 2.91 mW excitation power and 407 meV excitation energy, highlighting the fast decrease of the main lasing mode at 338 meV around 80 K. A spurious signal from the excitation laser at 352 meV is eliminated from the traces, for convenience. Raw data is given in the Supplementary Note 7. Inset: Ratio between all the lasing modes intensity (*I*_L_) and the total intensity (*I*_T_) for L5 as a function of the temperature for three different excitation energies. **b** Power emitted from the L5 samples as a function of the excitation power, for different temperatures

We furthermore investigate the temperature dependence of the laser characteristics. In Fig. 3a, emission spectra taken at 2.91 mW excitation power and energy of 407 meV are shown for various temperatures between 20 and 100 K. For temperatures near 80 K, the intensity of the main mode at 338 meV drops rapidly. Only some modes keep lasing at higher energy. At about 100 K, lasing altogether stops. The lower energy mode seems to suffer from upcoming parasitic absorption, most likely from the LH-HH transition. A similar gradual quenching of lasing modes from the low towards the high energy is observed in the roll-over regime for *E*_exc_ between 551 and 477 meV. Representative spectra are given in the Supplementary Note 3.

The inset of Fig. 3a shows the intensity of all the lasing modes, *I*_L_, compared to the total intensity *I*_T_. At lower excitation energy (407 meV) and lower temperature, nearly all the emission goes into the lasing modes (*I*_L_/*I*_T_ = 0.99). Above 80 K, the relative intensity of the lasing modes quickly decays. The quenching of lasing is remarkably abrupt, similarly to the temperature dependence of the first GeSn lasers, which turned off at ~90 K^11^. Here, however, the temperature at which lasing is quenched depends on the excess energy of the excitation and the strain applied: for excitation at 468 and 551 meV, the temperature at which *I*_L_/*I*_T_ < 0.5 decreases to 70 and 40 K, respectively. For the lower-strained sample L3 (not shown), lasing turns off at 65 K for 468 meV excitation. For bridges L1 and L2 with a strain lower than in L3, no lasing has been observed in spite of the fact that the spectra in steady state show a rich array of cavity modes. Figure 3b gives the LL curve for different temperatures, showing the transition from the ultra-high to a low efficiency regime and finally the dousing of lasing. In the following we like to elucidate this behaviour, which is not an universal feature for lasing in Ge-like systems—as we know from the case of GeSn where lasing is demonstrated already up to 230 K^38^—but, it applies for Ge systems near the direct to indirect band transition.

Indeed, the energy dependence of *T*_max_ does not agree with the one expected for the Shockley Read Hall (SRH) mechanism proposed to explain *T*_max_ for GeSn with rather low directness of Δ*E* = 25 meV^11^. Also, it is likely not due to thermal loading, which is found to be low (Supplementary Note 5). However, as we will argue, the remarkable sensitivity to excitation excess energy and the fast drop of *I*_L_/*I*_T_ hints at a non-equilibrium distribution between the population of the electrons in Γ and L, which is maintained over tens up to hundreds of picoseconds at low temperatures. The mechanism we propose is the blocking of phonon mediated Γ to L valley scattering.

### Γ to L valley scattering

**Fig. 4:**
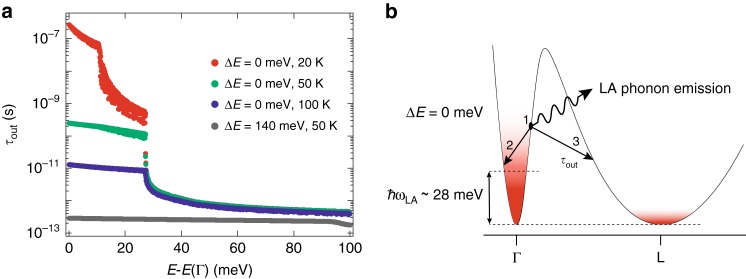
Tight-binding simulation of the phonon-assisted valley transfer time from Γ to L. **a** The latter is computed as the scattering time from an initial state in the Γ valley to final states out of the Γ valley. It is plotted as function of the energy above the bottom of the Γ valley at 50 K (grey) for the unstrained case when the offset (Δ*E*) between Γ and L is 140 meV; at 100 K (blue), 50 K (green), and 20 K (red) for the strained case with offset Δ*E* = 0 meV. The most effective scattering is due to the emission of a longitudinal (LA) and a transverse acoustic (TA) boundary zone phonons at energy *ħω* approximately of 28 and 10 meV, respectively. **b** Schematics of the conduction band with the Γ and L valleys aligned, i.e. Δ*E* = 0. After a carrier is optically excited in the Γ valley (1), it will relax within the Γ valley (2), or by the emission of a zone-boundary phonon—to establish momentum conservation—scatter into L (3). Carriers with energy < *ħω*_LA_ will remain much longer at Γ because the reverse process of absorbing a phonon is much less probable, in particular at low temperatures

In order to estimate the dynamics of electron scattering from Γ to L, the energy resolved phonon-assisted transfer time is calculated using the atomistic approach outlined in refs. ^39,40^. In Fig. 4a we show it at three temperatures, namely 20, 50 and 100 K, and compare the impact of the band structure when the offset Δ*E* between Γ and L is either 0 or 140 meV, which correspond to the here applied strained and the unstrained case, respectively. In the latter case, the transfer time is found to be very short, close to 200 fs, and almost independent on the energy of the electrons and the temperature (not shown), in excellent agreement with experiments^41,42^. However, at low temperature and in the strained configuration, electrons near the bottom of the band with kinetic energy beneath *ħω*_LA_ ~28 meV—which is the energy of the most relevant longitudinal-acoustic zone-boundary phonon—are essentially blocked, as shown in Fig. 4b. Above this phonon energy, the transfer time to L becomes fast again. The number of hot electrons transferred to L thus depends on the time they need to cool down to the lattice temperature, which may take several ps^41,43^. Similarly, at elevated temperature, the transfer rate increases due to the availability of thermally created phonons.

In spite of the oversimplifications made in the modelling of the valley transfer—namely, neither the e–e scattering nor the backscattering from L to Γ are included—several of the effects reported above can now be interpreted on a qualitative level: the distinct drop of lasing efficiency at around 80 K, the reduced *T*_max_ when the excess energy is increased or the strain is lowered, and the threshold dependence on the excess excitation energy are attributed to the growth of the L population together with the hole population and the concomitant increase in the intervalence band absorption. Moreover, the existence or absence of roll-over would be corresponding to a situation where the Fermi level *E*_F_ raises above or stays below the energy of the band offset plus *ħω*_LA_, respectively. Finally, as the L point energy is marginally below the one of the Γ valley, the lack of laser operation in continuous wave arises because of the slow but steady population build-up in the L minimum and its associated increase of intervalence band hole absorption, as the system must preserve charge neutrality. In contrast, the electron population in L remains negligible during the 100 ps of the pulsed operation, allowing efficient laser operation. In fact, if the direct configuration between Γ and L could be reached to an offset of just 20 meV, an electron concentration of 0.5 × 10^17^ cm^−3^—according to our calculation—would lead to a gain near 1000 cm^−1^, most likely sufficient to enable the lasing in steady state excitation and/or higher temperature operation. In fact, according to the here practiced tight-binding approach, an offset of 20 meV corresponds to a strain of 6.8%. While the current highest value at room temperature is 4.9%^24–26^, the theoretical ultimate strength is 15 GPa (14.6% of strain) in Ge along the <100> direction^44,45^. Reaching such values, for example at room temperature, is thus not a fundamental but rather a technical issue.

### Stimulated to spontaneous emission ratio

We finally would like to compare to the previous lasing reports with strained Ge^30,31^. As no power estimates is given in those works, we benchmark the ratio between cavity mode intensities and the spontaneous emission background, which relates to the ratio of radiative and non-radiative lifetimes, which we may assume is similar in the investigated structures. From Bao et al. we extract a maximal ratio of 4.5 (see Fig. 2a of ref. ^30^), which is definitely lower than our typical values for lasing which range from 10^1^ up to 10^2^ (see Supplementary Movie 1 and 2) but is higher than the ratio we obtain from the spontaneous emission of sample L5 shown in Fig. 2a, which is about 0.8. From Fig. 3 and Fig. S6 of the work of Elbaz *et al*.^31^ we extract a ratio of 0.15. Because this value taken from^31^ is obtained for the case of TM modes in a micro disc cavity arrangement, we thus compare it to the lasing demonstration in GeSn microdiscs^46^ where (see Fig. 3 of ref. ^46^) a ratio of about 30 is reached, independent on the polarisation. Indeed, the spectra shown in refs. ^30,31^ resemble much more the above shown (amplified) spontaneous emission spectra obtained in CW excitation than any of the here and elsewhere reported lasing spectra.

## Discussion

Lasing was achieved in strained Ge microbridges integrated in corner-cube cavities under 100 ps excitation at energies near the strained Ge bandgap. We showed clear transition from spontaneous to stimulated emission (see Supplementary Movie 1), threshold at low carrier concentration below 10^17^ cm^−3^, narrow lines (<0.1 meV) and orders of magnitude increase of the emission efficiency up to a value that corresponds to a differential quantum efficiency of above 50%. Supported by our theoretical modelling, we argue that the strain in our samples—in contradiction with most of the previous predictions—provides a direct bandgap configuration only marginally; high gain and lasing is achieved thanks to a blocking at low temperature of phonon-assisted valley scattering, which keeps the electron in the Γ valley and thus provides high gain. In order to increase the lasing operation temperature, the Γ to L offset has to become larger, as we know from the published GeSn results. But, even supposing that the technological node of higher strain is mastered, the upper bound of lasing operation temperature will hardly reach 300 K. In fact, the emission energy will shift into the long wavelength mid-infrared range (>3 µm) where room temperature operation is traditionally reached only by the unipolar quantum cascade laser^47,48^ or by the interband cascade laser^49^. However, our study pinpoints the investigation of alternative proposals to reach room temperature operation, like the combination of strain and n-doping^34^, as well the electrical injection, which is similar to the near resonant optical pumping, which we showed is highly favourable. Moreover, the electrical injection comes with the further advantage, respect to the optical pumping, to control the holes and electron density separately, or to remove the accumulated carriers continuously or sequentially under reverse-biased, similarly as shown in ref. ^50^, enabling lasing operation at higher temperatures.

## Methods

### Microbridge fabrication

Starting from a germanium-on-insulator (GeOI) substrate consisting of an undoped and slightly biaxially pre-strained (0.16%) Ge layer of 1 µm over a buried 1 µm SiO_2_ layer^51^, highly strained Ge microbridges are fabricated with an integrated optical cavity. Using e-beam lithography and reactive ion etching, we define two stressor pads connected by a constriction, and two parabolically shaped mirrors forming the optical cavity. By removing the underlying SiO_2_ layer using HF vapour etching, a relaxation of the stressor pads and thus a stretch of the bridge occurs along the crystallographic direction <100>. The tensile strain is determined solely by the geometrical dimensions of the pattern (see Supplementary Note 6). For all L3 to L5 cavities the gain material has the following volume of 8 × 1 × 1 µm^3^.

### Spectroscopy set-up

Samples were mounted in a He flow cryostat using a clamping system to avoid glue solvent near the sample. They were excited using either a continuous wave semiconductor diode laser from Brolis operating at 590 meV, or an optical parametric oscillator (OPO) from EKSPLA, enabling pulsed excitation (100 ps) at 83 MHz repetition rate and continuous tuning in the 370–551 meV energy range. The excitation light is absorbed uniquely by the strained microbridge, as the relaxed Ge of the pads is transparent for the explored energy range. Excitation powers are given as time average and integrated over the area of a 10 µm diameter pinhole.

Both laser excitation as well as the collection of the emitted light occurs via a reverse Cassegrain reflection microscope objective (Newport) with a ×15 magnification and a numerical aperture (NA) of 0.4. The emission is collected in the direction normal to the sample surface, and then guided through an FTIR spectrometer (Bruker Vertex) that run either in step-scan or fast-scan configuration, providing a non-apodized spectral resolution of 62 µeV. The emission is detected by a liquid nitrogen cooled InSb photodiode (PD) equipped with a customized cold shield to reduce NA and thus the thermal background. The signal from the diode is amplified by 10^8^ V/A before read-out by a Lock-in amplifier (in step-scan configuration) and recorded by an analogue to digital converter (ADC). The sensitivity of the optical set-up inclusive the detector and amplifier was calibrated against a thermal source, as reported in the Supplementary Note 8, enabling us to report directly the power of the emitted light collected by the microscope.

## Supplementary information

Supplementary Information

Peer Review File

Description of Additional Supplementary Files

Supplementary Movie 1

Supplementary Movie 2

## Data Availability

The data that support the findings of this study are available upon request to the corresponding.
