# Supplementary material for: Lasing in strained germanium microbridges
Source: Nat Commun. 2019 Jun 20;10:2724. doi: 10.1038/s41467-019-10655-6 (PMC6586857; doi:10.1038/s41467-019-10655-6)
Supplement: Supplementary file 1 — Supplementary Information [file 41467_2019_10655_MOESM1_ESM.pdf]

Supplementary Information: Lasing in strained germanium microbridges

F.T. Armand Pilon[1,4+], A. Lyasota[1], Y-M Niquet[3],
V. Reboud[2], V. Calvo[3], N. Pauc[3], J. Widiez[2], C. Bonzon[4], J.M. Hartmann[2], A. Chelnokov[2],
J. Faist[4] and H. Sigg[1+]

[1] Laboratory for Micro- and Nanotechnology, Paul Scherrer Institut, 5232 Villigen, Switzerland
[2] Univ. Grenoble Alpes, CEA, LETI, 38054 Grenoble, France
[3] Univ. Grenoble Alpes, CEA, IRIG-DePhy, 38054 Grenoble, France
[4] Institute for Quantum Electronics, ETH Zürich, 8093 Zürich, Switzerland

+email: hans.sigg@psi.ch; francesco.armand-pilon@psi.ch

Supplementary Note 1: Mode shift analyses

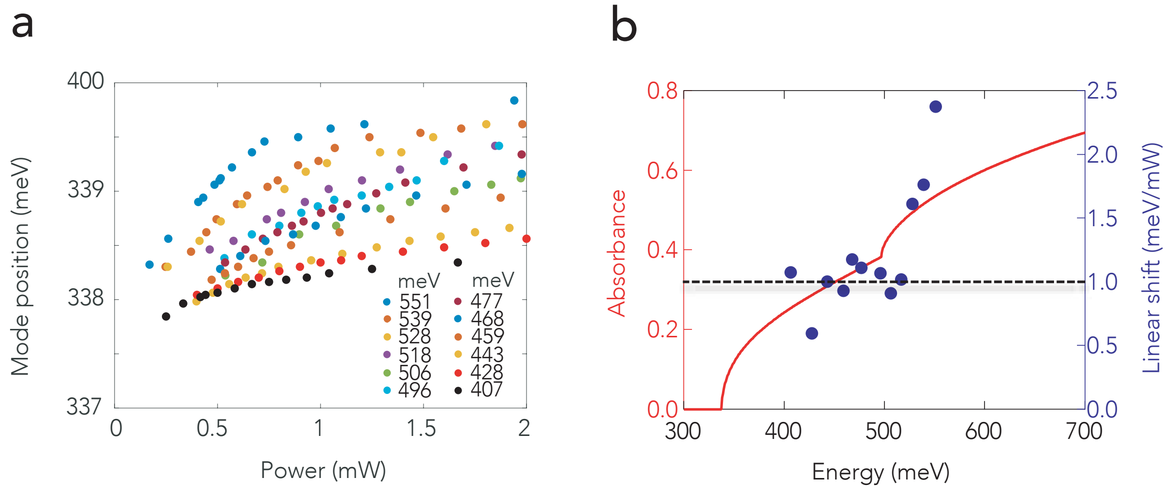

Supplementary Figure 1 | Shift of the main lasing mode of the L5 microbridge at 20 K. a, Experimental shift of the main lasing mode position in function of the average excitation power for different excitation energies, used to estimate the **b,** carrier concentration dependence on the excitation energy.

Supplementary Figure 1a shows the experimental position of the main lasing mode of the L5 microbridge measured in pulse regime at 20 K at excitation energies ranging from 407 to 551 meV as a function of the excitation power up to 2 mW. Starting from 337.6 meV, the mode blue shifts linearly, followed by a sublinear increase, for excitation powers of above about 0.5 mW. Supplementary Figure 1b gives the slopes of the mode shift extracted from a linear fit to the data taken at low intensity of Supplementary Figure 1a, superimposed to the absorbance, i.e. attenuation calculated for a 1 µm thick Ge layer with 6% strain. The mode shift reflects the change of refractive index which relates to the density of injected carriers [1]. We make use of this correlation to estimate the density of excited carriers by "calibrating" the slope respect to one excitation energy. We use the 551 meV excitation as the reference, since the corresponding absorbance value is the highest, and thus, the interference effects are expected to be the smallest. In this case, we simply estimate the carrier density from the energy we dump per pulse in the 8x1x1 μm^3 volume (V) of the bridge. For an average power of 1 mW– as measured through the pinhole of 10 µm diameter at the sample position – we thus obtain 0.34 pJ, i.e. about 3.8 x 10^6 photons. Hereby we consider that: only about 10 % of the incident light hits the bridge, 65 % of the excitation is lost by reflection at the Ge/air surface and the cryostat window and each of the remaining photons have a 0.8 probability of being absorbed. This value is larger than the calculated film absorbance of 0.55, as reported in Fig.A1b, because we take also internal reflection into account. The resulting number of excited carriers per 1 mW corresponds to a density of about 4.8 x 10^{17} cm^{-3}, i.e. 1 carrier excited per about 3.6 photons impinging the bridge, bringing the quantum pumping efficiency to 28 %. For each excitation energy lower than 551 meV, we first determine from Supplementary Figure 1b the ratio of its slope value with respect to the reference one, namely 2.4 meV/mW. In particular, for all the energies below 518 meV we consider an average slope value of 1 meV/mW, as indicated in Supplementary Figure 1b. We then estimate the carrier density as the fraction of the reference density corresponding to the slope ratio. For the case of 407 meV, the thus extracted carrier concentration for 1 mW is (1/2.4) x 4.8 x 10^{17} = 2.0 x 10^{17} cm^{-3}, which – as a simple calculation

shows – corresponds to a quantum pumping efficiency of 8.6 %, namely 1 carrier is excited per about 12 photons impinging on the bridge.

Supplementary Note 2: Spectral and linewidth evolution of the main lasing mode of L5

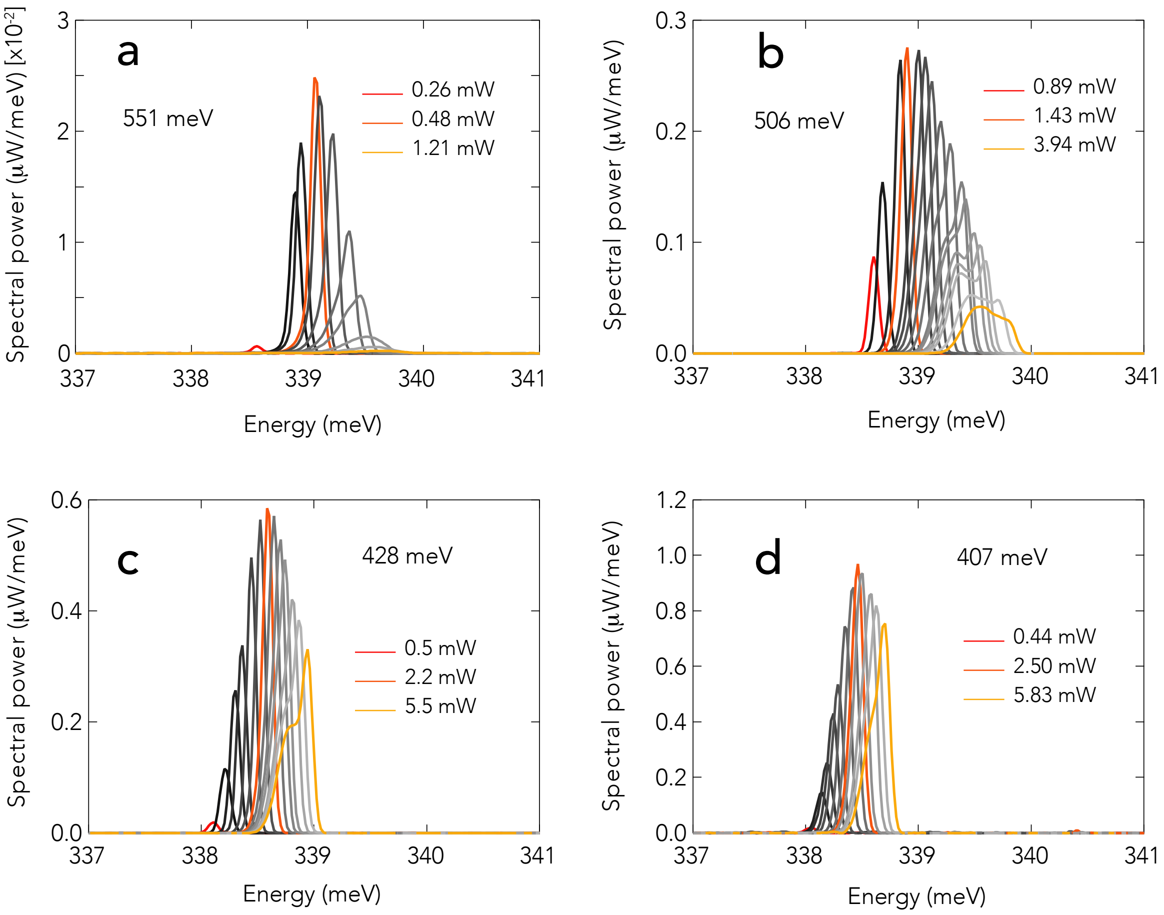

Supplementary Figure 2 | Spectra evolution of the L5 main lasing mode at 20 K. Spectra taken from low to high power with excitation energy of **a,** 551 meV, **b,** 506 meV, **c,** 428 meV and **d,** 407 meV.

Here we address the dependency of the integrated output power on the excitation energy shown in Fig. 2b (main text), as well as the linewidth power dependence shown to increase for the highest powers, in Fig. 2e, (main text). In Supplementary Figures 2a, b, c and d, we provide spectra of the main lasing mode of L5 taken at 20 K for E_{exc} = 551, 506, 428 and 407 meV, respectively. In each plot, three values of the excitation power are highlighted: the lowest value at which we have a distinct mode, the value at which the mode reaches its highest peak intensity, and the one obtained at the highest excitation power of this measurement series, or, for the 551 meV case, the last spectrum before the lasing of the main mode has completely quenches. In all cases, the linewidth of the mode remains narrow up to the peak maximum. From then on, the line broadens. For E_{exc} = 551 and 506 meV, the integrated mode intensity also decreases, resulting in a roll-over of the total integrated intensity. For the lower excitation energies reported, the integrated intensity keeps sub-linearly increasing.

Supplementary Note 3: Extended view of the lasing spectra for L5

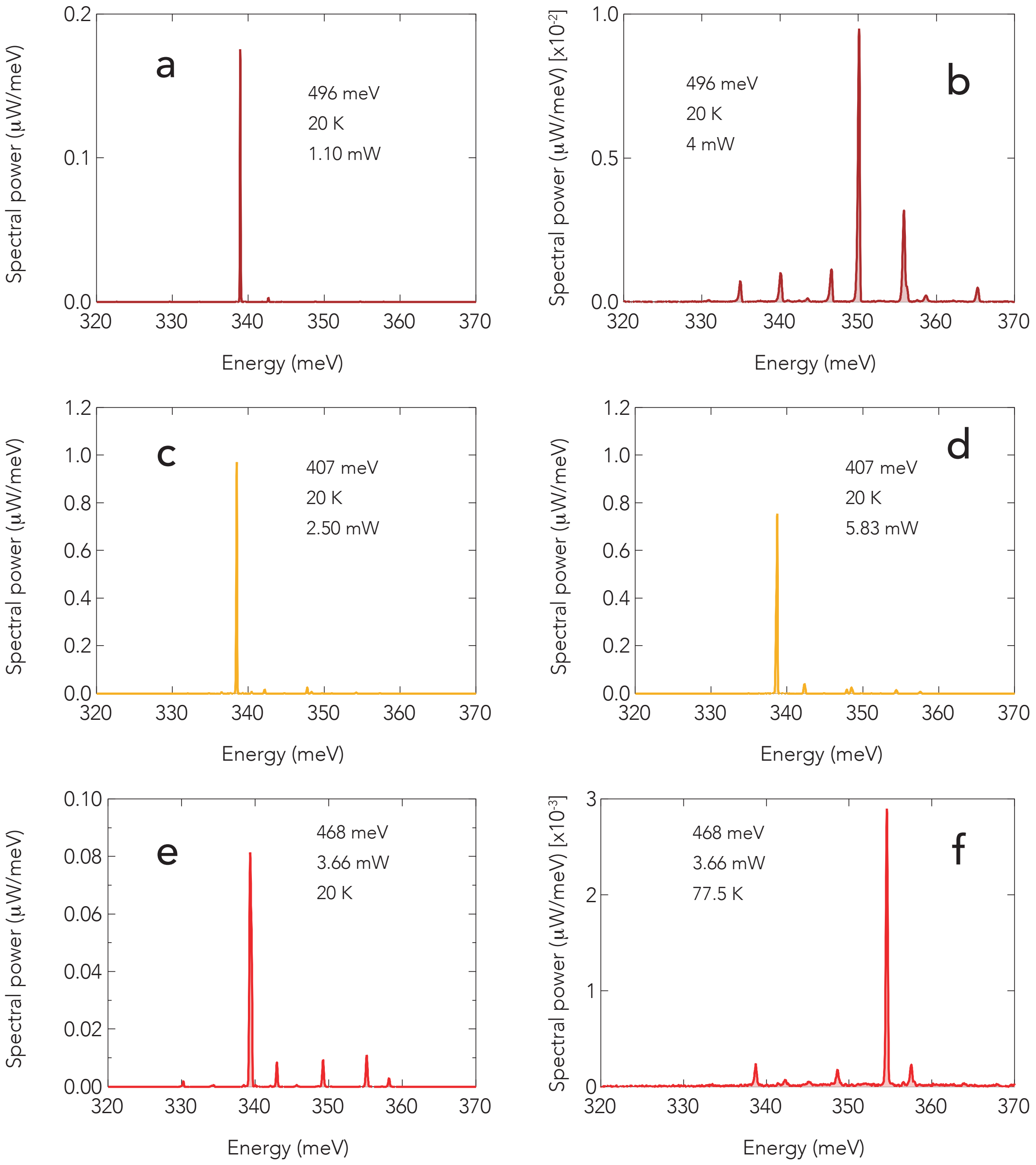

Supplementary Figure 3 | Overall view of the lasing spectra for L5. We compare the medium and high power, as well the low and high temperature, lasing spectra for the L5 microbridge. Near single mode spectra at 20 K are shown in **a,** for E_{exc} = 496 meV, **c,** E_{exc} = 407 meV and **e,** E_{exc} = 468 meV. The high power spectra are reported for excitation power of **b**, 4 mW and **d,** 5.83 mW, while the spectrum at high temperature is shown in **f**, at 77.5 K.

In this section, we focus on the overall picture of lasing, and include in addition to the main lasing mode, those at higher energies. In particular, we want to highlight the difference in lasing spectra when the power is equal to or higher than the value at which the main lasing mode reaches its highest intensity. For E_{exc} = 496 meV, the main lasing mode strongly collapses when the power is increased up to the roll-over regime, see Supplementary Figures 3a and b.

Remarkably, starting from a single mode spectrum, the lasing evolves toward a multimode configuration. In the 407 meV case, the spectrum is by contrast close to single mode up to the maximum power, see Supplementary Figures 3c and d. The increase in intensity of the modes at high energy and the broadening of the main lasing mode help sustaining the continuous increase in the total integrated intensity. Finally, we investigate the temperature dependence of the modes excited at E_{exc} = 468 meV. In Supplementary Figures 3e and f, we show spectra at 20 K and 77.5 K (same power). At high temperature, similarly to the high intensities for the high excitation energy, there is a strong quenching of the main lasing mode, in favour of a multiple of higher energy modes.

Supplementary Note 4: Single mode lasing

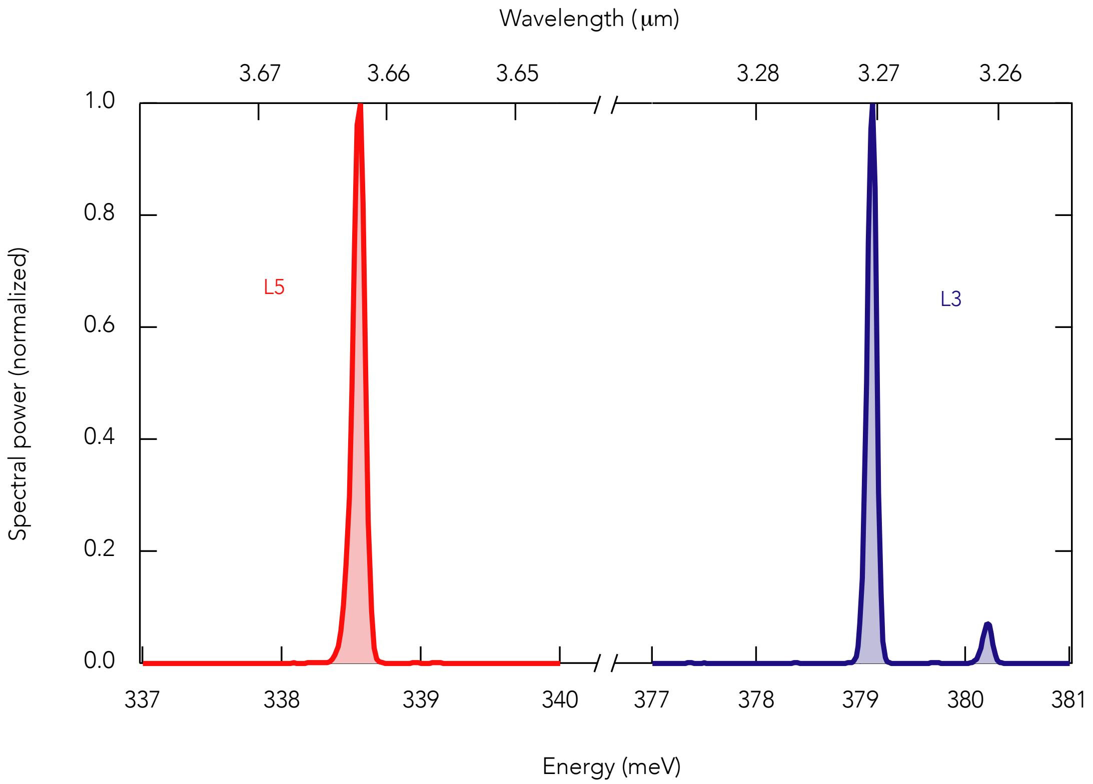

Supplementary Figure 4 | Magnified view of L5 and L3 lasing spectra. Spectra of the L5 and L3 microbridges at 20 K, excited at 428 meV energy with an average power of 2 mW.

Supplementary Figure 4 shows on a magnified *x*-axis the lasing spectra of L5 and L3 samples upon pulsed excitation, reported in the Fig. 2a, (main text). The spectra are normalized and show an almost perfect single mode emission. The linewidth (FWHM) of 0.135 and 0.105 meV for L5 and L3, respectively, are near to the spectral resolution of the spectrometer. The spectra reported here contrast the PL spectra, where the emission is multimode and covers an energy range of about 100 meV, highlighting the strong mode competition of the lasing action.

Supplementary Note 5: Thermal Considerations

Using the same approach described in the Supplementary Note 1, we deduce that the energy absorbed by the bridge for a 1 mW average power at $E_{exc} = 551$ meV is about 0.3 pJ. The local temperature of the bridge will therefore raise, at 20 K, by about 0.5 K, as obtained from $\Delta T = Q/(c_p\,\rho\,V)$, where c_p is the thermal capacity and ρ the material density. Thanks to the high thermal conductivity of Ge (> 5 W cm^{-1} K^{-1} at $T < 50$ K [2]), the system cools down effectively between two pulses from the OPO, and the equilibrium temperature at 1 mW incidence is very close (0.1 K) to the base temperature, indicating that thermal loading is amazingly low for this experiment.

Supplementary Note 6: Deterministic Strain determination in microbridges

**Sample**	**Pad length *B* (*μ*m)**	**EF w/o cavity**	**ε_{100} (%) @300 K**	**ε_{100} (%) @20 K**	**Raman shift Δ*ω* (cm^{-1}) w/o cavity**	**ε_{100} (%) from Raman shift**
L1	186	20.15	3.10	5.04		
L2	212	21.99	**3.38**	5.50	6.07	**3.38**
L3	240	23.85	3.66	5.97		
L4	260	25.11	**3.85**	6.28	6.96	**3.74**
L5	280	26.32	4.05	6.60		
L6	300	27.47	4.22	6.87	-	-

Supplementary Table 1 | Geometrical dimension of samples from L1 to L6 without cavity and the corresponding determined strain values, at 300 and 20 K. The strain values obtained from the Raman measurement at 300 K are also provided for comparison purposes.

**Sample**	**Pad length *B* (*μ*m)**	**EF with cavity**	**ε_{100} (%) @300 K**	**ε_{100} (%) @20 K**	**Raman shift Δ*ω* (cm^{-1}) with cavity**	**ε_{100} (%) from Raman shift**
L1	186	18.13	2.78	4.54		
L2	212	19.80	**3.04**	4.96	5.24	**3.00**
L3	240	21.46	3.30	5.37		
L4	260	22.60	**3.47**	5.66	5.90	**3.30**
L5	280	23.68	3.64	5.93		
L6	300	24.72	**3.80**	6.19	6.95	**3.74**

Supplementary Table 2 | Geometrical dimension of samples from L1 to L6 with cavity

The strain in suspended germanium-on-insulator (GeOI) based microbridges depends on basically two parameters; (a) the built-in strain of the Ge layer ε_0 and (b) the enhancement factor (EF) by which this pre-strain is enhanced. The pre-strain depends on the fabrication processes of the GeOI [3], while EF depends on the geometry of the bridge structure. We determine the strain at room temperature by Raman scattering, for a selected number of bridges, from which we obtain accurate strain values for the full set of samples and various temperatures. Details of the procedure can be found in [4], [5] and in the following paragraph:

A microbridge geometry is defined by the bridge length (A) and width (a), the total pad length (B) and width (b), as well as the additional under etching of the pads (L). The latter is basically the only processing related parameter. Typically, only one of the parameters is varied in a series, namely the pad length, whereas all other parameters stay the same. For the example reported in Supplementary Table 1 and 2, which refer to the series here employed, the dimensions A, a and b are set to 8, 1 and 80 μm, respectively. We take into account an additional under etching L of 10 μm. The geometrical enhancement factor EF follows from the equation from Ref [6].

$$\mathrm{EF} = \frac{2L + B}{B}\left(\frac{A}{B - A} + 1\right) / \left(\frac{A}{B - A} + \frac{a}{b}\right)$$

For the series with pad length L1 to L6 we obtain the values given in Supplementary Table 1. Next, we determine the strain from the Raman shift - using the non-linear conversion model as described in Ref [7] - on a subset of samples which include bridges with and without cavities. In the present case, we could reproduce the experimental values in the model by postulating that $\varepsilon_0 = 0.154$ %, which is very close to the value obtained by x-ray diffraction, i.e. $\varepsilon_0 = 0.16\%$. The experimental strain values for samples with cavity are reduced by about 10 % compared to that in identical microbridge structures without cavity. The reduction factor of approximately 10 % is in good agreement with COMSOL simulations and translates into a reduced effective EF, as reported in Supplementary Table 2.

In Supplementary Figure 6, we report the PL spectra of an L6 sample with cavity for various temperatures. The increase of the pre-strain value ε_0 as the temperature decreases is parametrized with the following function which fits the thermal expansion coefficients given in [8],[9] for Ge and Si, respectively:

$$\begin{aligned}\frac{\varepsilon_0(\mathrm{T})}{100} = &-2.65466 \times 10^{-4}\exp[-0.00588\, T_0] + 2.65466 \times 10^{-4}\exp[-0.00588\, T] \\ &+ 2.298 \times 10^{-6}(T_0 - T) + 1.5226 \times 10^{-9}({T_0}^2 - T^2) \\ &- 1.16667 \times 10^{-13}({T_0}^3 - T^3)\end{aligned}$$

T and T_0 are in units of Kelvin, where T_0 refers to the temperature at which the Ge layer is relaxed. The latter, for the GeOI here used, is found to be T_0 = 701 K [4], and it typically corresponds to the growth or the subsequent annealing temperature of the fabrication process. We can observe a strong red shift of the photo luminescence emission, which reflects the narrowing of the band gap at Γ. From the tight binding (TB) model [10] and taking into account the Varshni shift [11], [12] the nominal position of the band gap is obtained, see black lines in Supplementary Figure 6. We observe a good agreement of experiments with modelling, down to 100 K. However, when the temperature is further decreased, the PL emission does not follow the model systematically, as the two 20 K light blue spectra, both for L6 bridges, show. This effect is predominately observed for structures with cavities. In this case, the strain is interfered from the position of the PL spectrum. Unfortunately, none of the L6 samples with cavity survived until the start of the OPO measurements.

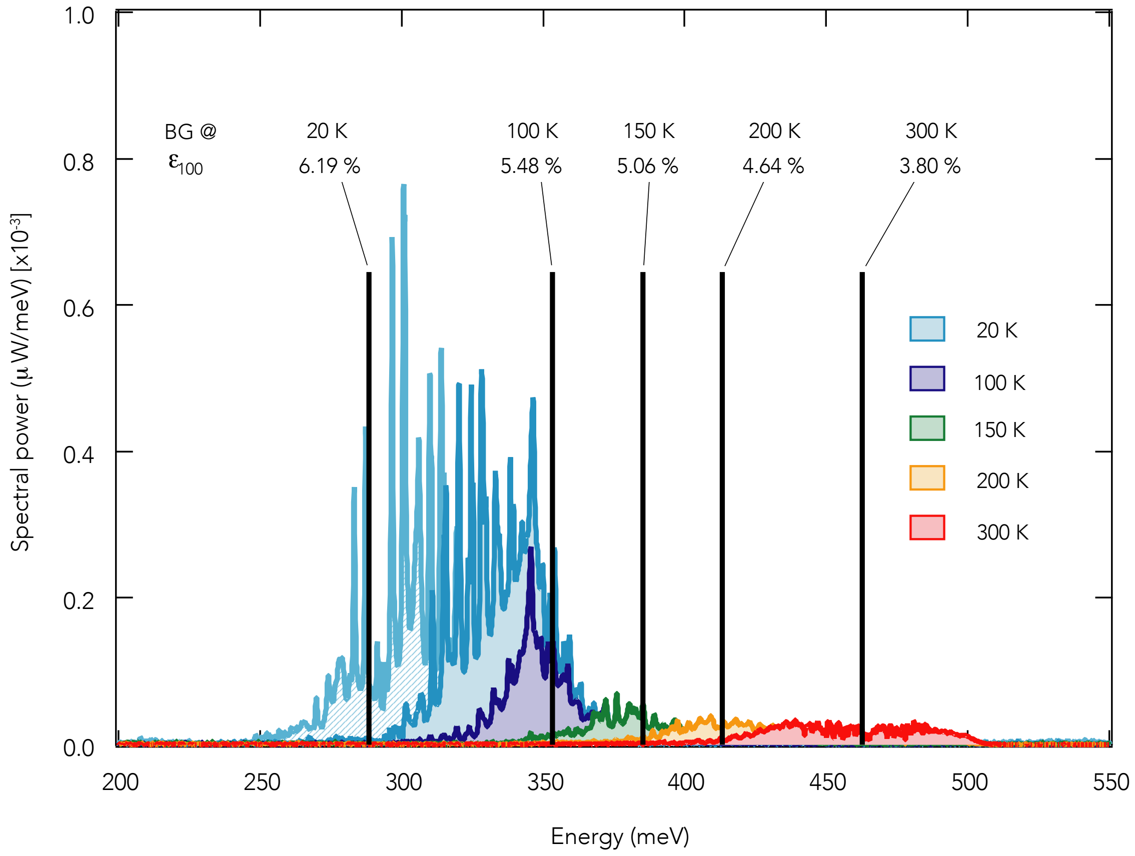

Supplementary Figure 6 | Red shift of the PL spectra of the L6 sample with cavity in function of the temperature. For each temperature, the nominal position of the band gap is reported. All the spectra are obtained with an excitation energy of E_{exc}= 590 meV in steady state.

Supplementary Note 7: Determination of the spurious signal power

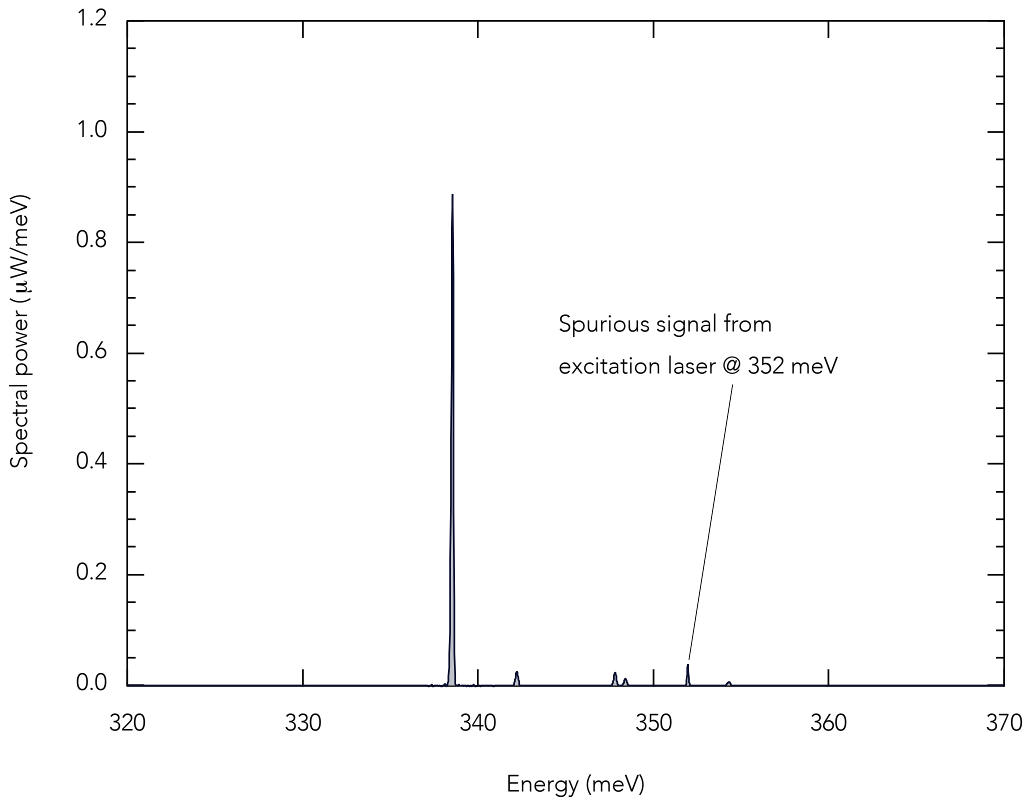

Supplementary Figure 7 | Spurious signal near resonant excitation. Lasing spectrum of L5 microbridge at 20 K, excited at 407 meV energy with a power of 2.91 mW**.**

Supplementary Figure 7 is intended to show the spurious signal at 352 meV which is eliminated from the traces in the Fig. 3a of the main text, for convenience. It is a non-linearly generated line from the OPO laser of low power which is reflected from the sample surface. We estimate the power of the incoming spurious signal to be 0.15 μW. As the lowest threshold value found in the experiment is 10^3 times higher, the spurious signal is not affecting the measurement.

Supplementary Note 8: Estimation of the quantum efficiency

Corner-cube mirror concept: The principle of operation of the corner-cube cavity is similar to a Fabry-Perot etalon, as it is formed by two mirrors reflecting the radiation backwards to the gain region. Each of the two reflectors consists of a pair of parabolic mirrors successively collecting and refocusing the radiation coming from the strained bridge waveguide back into the bridge. A total of four reflections under the critical angle of total internal reflection, yield a very high mirror reflectivity. The two reflector regions are located in the pad which is almost strain free and are thus transparent to the operation energy. Unlike DFB or VCSEL cavities, this cavity is almost free of dispersion and is therefore inherently multimode and tailored to a strained laser gain medium whose gain peak varies strongly with the geometrical size of the bridge and the pads, as well with the temperature. Furthermore, its process can be integrated in

the same fabrication layer as the rest of the structure and the reflectors are thus well aligned to the bridge by design.

Cavity Emission: The TE-like fundamental mode field of a Ge waveguide with 1μm width and 1μm height suspended 1μm above a Si substrate (see Supplementary Figure 8a), is calculated by the 3D finite element method of the RSOFT Suite. The effective refractive index of the TE-like fundamental mode at 354 meV energy is found to be equal to 3.58. The reflection from a single corner cube is "measured" by the back reflected power of the fundamental mode field "launched" from the centre position of the microbridge. The optical propagation is calculated by the 3D FDTD of the RSOFT Suite to be 90.6%, which corresponds to the cavity loss per round trip is equal to 18 % when no additional losses, like scattering from the sidewalls or internal absorption channels, are taken into account. Placing a power detector, with a surface of 1600 μm^2, 1 μm above the Ge surface we obtain about 2.8 % of the power is scattered out of plane in the direction of the optical set up per round trip, which correspond to 15 % of the total optical loss. The far-field intensity distribution for the whole Ge optical cavity composed of two corner cubes is reported in Supplementary Figure 8b. By its integration within the solid angle covered by the microscope objective, whose numerical aperture (NA) is 0.4, the collection efficiency of the Cassegrain is calculated to be 9.1 %. For the latter, the shadow cone within NA = 0.2 of the microscope objective is considered.

Set-up calibration: The quantum efficiency of the lasing L5 microbridge for the case of excitation at 407 meV is estimated as follows. We first calibrate the set-ups efficiency by measuring in an identical configuration and photon energies as used here, the signal of a black body emitter of known emissivity, temperature and size, placed at the sample position. Assuming a Lamberts emission pattern, the quantum conversion factor at the photon energy of around 350 meV is determined to be about 4 %. This number includes the detectors quantum efficiency of about 50% and enables to convert the detected signal at the output of the signal amplifier, set with a gain of 10^8 V/A, to the collected power: for the here discussed case we read for an average input power of 1 mW, a signal of 655 mV, which correspond to a power of 0.055 μW at the exit of the microscope (see Fig. 2b of the main text). This, with the above calculated collection and out-couple efficiencies, converts to a total of outward (in plane and out-of-plane) radiated intensity of 0.055 μW/(0.15 x 0.091) = 4.03 μW. On the other hand, an input power of 1 mW, measured through the 10 μm pinhole at the sample position, corresponds to 100 μW impinging on the surface of the bridge of area 8 μm^2. When using the previously determined absorption of only 1 per 12 in-pinging photons (see Supplementary Note 1), we obtain that the power absorbed in the bridge is about 8.62 μW. The slope of the curve so obtained hints the systems efficiency in terms of energy. For the here analysed excitation of 407 meV, we extract a value of 80 %, corresponding to a differential quantum efficiency, namely the ratio of the number of photon s^{-1} radiated from the cavity with respect to the number of photon s^{-1} absorbed, close to 100%. The accuracy of this number is determined by mainly the uncertainty (about a factor of 2) in the conversion of detector signal to collected power. A second source of uncertainties comes from the accuracy by which the sample is oriented with its normal towards the Cassegrain optics, which is relevant because of the serious shadowing of light traveling on-axes. Thirdly, we systematically neglect any scattered light from potentially rough surfaces. Since the last two effects give an opposite contribution and moreover are expected to be less significant than the first one, we estimate the lower bound of the differential quantum efficiency to be 50%.

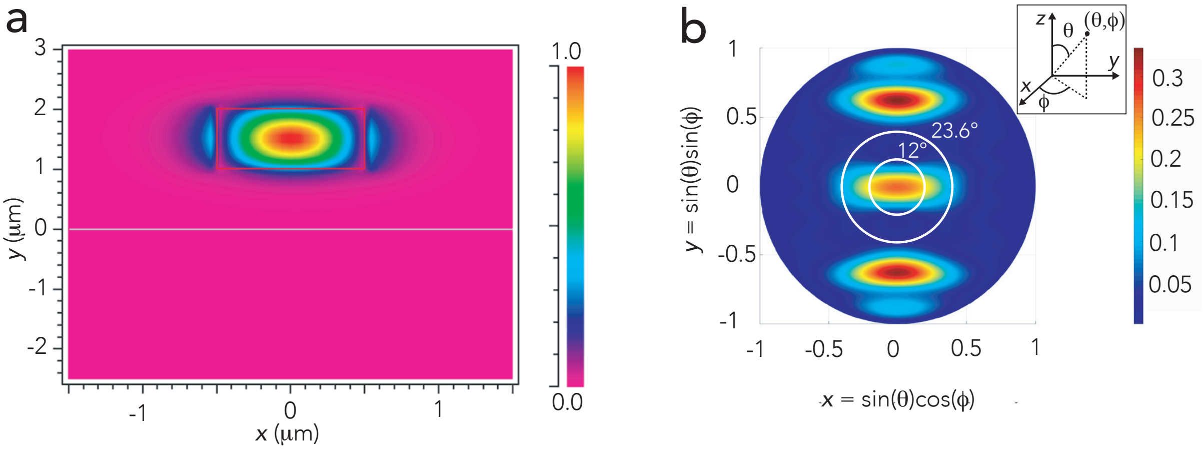

Supplementary Figure 8 | Simulation of the light confinement in the microbridge and its emission from the cavity. a, TE fundamental mode field distribution in a Ge microbridge with 1 μm width and suspended 1 μm above a silicon substrate. **b**, Polar projection of the far field intensity distribution of the light emitted from a corner cube cavity with a length of 44 μm. The light is mainly emitted in 3 direction: (θ,ϕ) = (± 40°, 90°) and (θ,ϕ) = (0°, 0°). The latter corresponds to the direction perpendicular to the surface. The two white circles indicate the θ angles within the light is effectively collected by the objective, namely 12 °and 23.6 °. Inset: the spherical coordinate system here adopted.

Supplementary Note 9: Strained germanium band structure

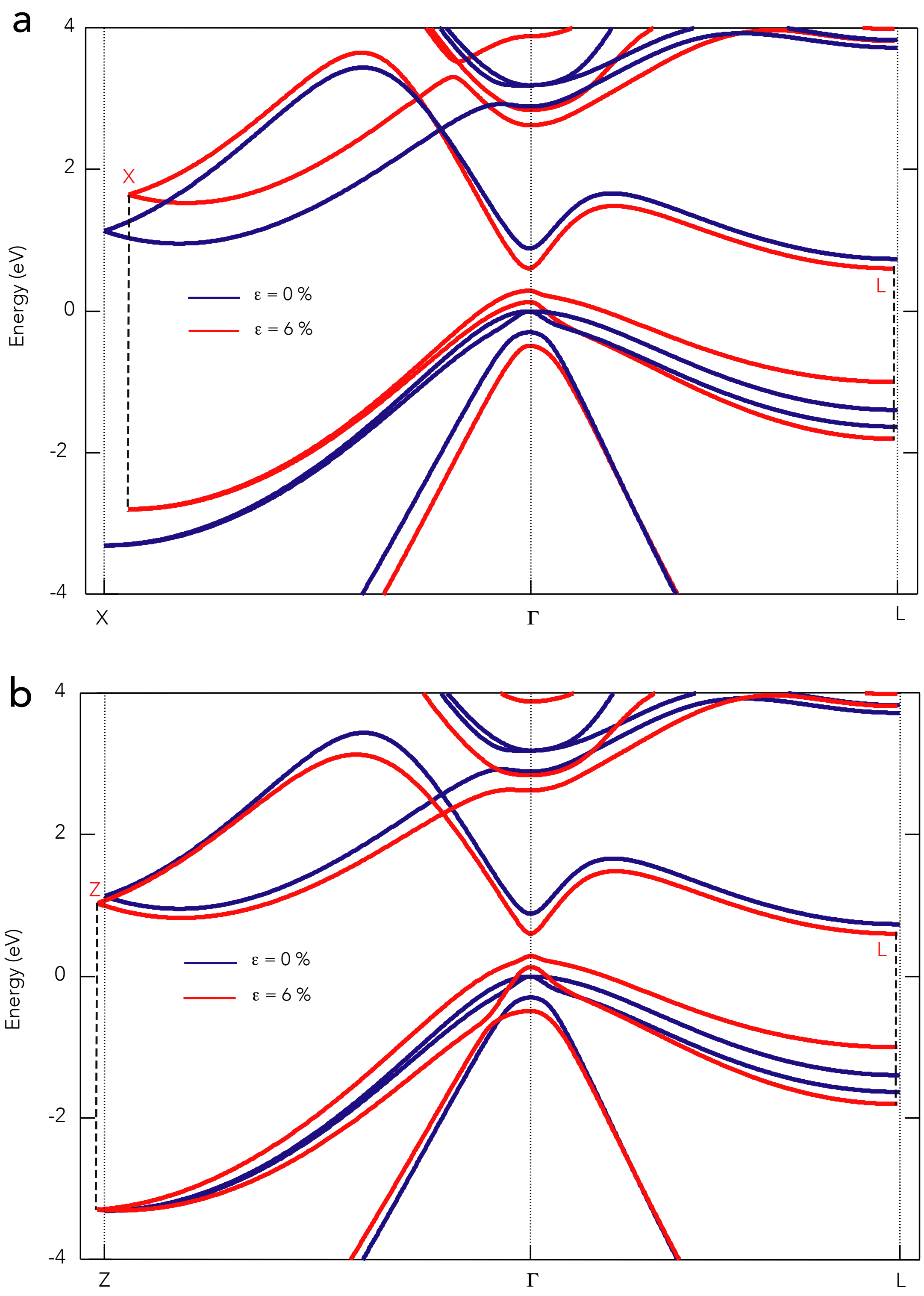

Supplementary Figure 9 | Band structure of germanium. Using the tight-binding model, the band structure of germanium is computed at 20 K under 6 % (red) and 0 % (blue) of strain along the [100] direction, along the path **a,** from points X to Γ then L, and **b,** from points Z to Γ to L of the first Brillouin zone (FBZ). For both paths, the dashed (dotted) lines are positioned in correspondence with the symmetry points in the strained (unstrained) FBZ. Under strain the absolute distance in **k**-space from Γ to X (in **a**) and to Z (in **b**) decreases and increases, respectively, as shown by the shift of the dashed lines with respect to the dotted ones.

We report here the band structure of germanium, computed with the tight-binding model as detailed in the main text. Supplementary Figure 9 highlights the evolution of the band structure going from 0 to 6 % of uniaxial strain along the [100] direction. In particular, we show the band structure from Γ to L, whose symmetry is conserved under the uniaxial strain, and from Γ to both the X, see Supplementary Figure 9a, and Z valleys, see Supplementary Figure 9b, whose symmetry is broken by the applied strain (X and Z being aligned along and perpendicular to the [100] direction, respectively). With a loading of $\varepsilon = 6$ %, in line with our experiments, the band structure approaches the direct band gap crossing. Moreover, the strain lifts up the degeneracy of the top two valence bands (light and heavy hole). Evidentially, the transition of the light to heavy hole at Γ is smaller than the band gap, while the splitting to the split-off (SO) band is larger, ensuring low parasitic intervalence band absorption of free holes, as the low lasing threshold values indicate.

Supplementary References

[1] R. Geiger *et al.*, “Excess carrier lifetimes in Ge layers on Si,” *IEEE Int. Conf. Gr. IV Photonics GFP*, vol. 062106, pp. 103–104, 2013.

[2] H. M. Rosenberg, “The thermal conductivity of germanium and silicon at low temperatures,” *Proc. Phys. Soc. Sect. A*, vol. 67, no. 9, pp. 837–840, 1954.

[3] V. Reboud *et al.*, “Structural and optical properties of 200 mm germanium-on-insulator (GeOI) substrates for silicon photonics applications,” vol. 9367, no. i, p. 936714, 2015.

[4] T. Zabel *et al.*, “Top-down method to introduce ultra-high elastic strain,” *J. Mater. Res.*, vol. 32, no. 4, pp. 726–736, 2017.

[5] R. A. Minamisawa *et al.*, “Top-down fabricated silicon nanowires under tensile elastic strain up to 4.5%,” *Nat. Commun.*, vol. 3, no. May, 2012.

[6] M. J. Süess *et al.*, “Analysis of enhanced light emission from highly strained germanium microbridges,” *Nat. Photonics*, vol. 7, no. June, pp. 466–472, 2013.

[7] A. Gassenq *et al.*, “Accurate strain measurements in highly strained Ge microbridges,” *Appl. Phys. Lett.*, vol. 108, no. 24, 2016.

[8] H. P. Singh, “Determination of thermal expansion of germanium, rhodium and iridium by X-rays,” *Acta Crystallogr. Sect. A*, vol. 24, no. 4, pp. 469–471, 1968.

[9] Y. Okada and Y. Tokumaru, “Precise determination of lattice parameter and thermal expansion coefficient of silicon between 300 and 1500 K,” *J. Appl. Phys.*, vol. 56, no. 2, pp. 314–320, 1984.

[10] Y. M. Niquet, D. Rideau, C. Tavernier, H. Jaouen, and X. Blase, “Onsite matrix elements of the tight-binding Hamiltonian of a strained crystal: Application to silicon, germanium, and their alloys,” *Phys. Rev. B - Condens. Matter Mater. Phys.*, vol. 79, no. 24, 2009.

[11] Y. P. Varshni, “Temperature Dependence of the Energy Gap in Semiconductors,” *Physica*, vol. 34, pp. 149–154, 1967.

[12] C. D. Thurmond, “The Standard Thermodynamic Functions for the Formation of Electrons and Holes in Ge, Si, GaAs, and GaP,” *J. Electrochem. Soc.*, vol. 122, no. 8, p. 1133, 1975.
