# Supplementary material for: Lasing in strained germanium microbridges
Source: Nat Commun. 2019 Jun 20;10:2724. doi: 10.1038/s41467-019-10655-6 (PMC6586857; doi:10.1038/s41467-019-10655-6)
Supplement: Supplementary file 3 — Description of Additional Supplementary Files [file 41467_2019_10655_MOESM3_ESM.pdf]

Description of Additional Supplementary Files

File Name: Supplementary Movie 1
Description: Power dependence spectra of L4 sample at 20 K under pulsed excitation energy of Eexc = 551 meV, in a linear (left panel) and logarithmic scale (right panel). The legends report the time averaged excitation power, integrated over the area of a 10 μm diameter pinhole. The spectra are acquired in step-scan configuration, highlighting the evolution from a multimode spontaneous emission below threshold to a clear lasing action of just few cavity modes.

File Name: Supplementary Movie 2
Description: Power dependence spectra of L5 sample at 20 K under pulsed excitation energy of Eexc = 443 meV, in a linear (left panel) and logarithmic scale (right panel). The legends report the time averaged excitation power, integrated over the area of a 10 μm diameter pinhole. The spectra are acquired in fast-scan configuration. The logarithmic scale highlights the steep increase of the lasing cavity modes height with respect to the background up to about 4 order of magnitude, as the pumping energy approaches the resonance condition.
