# Supplementary material for: Lasing in strained germanium microbridges
Source: Nat Commun. 2019 Jun 20;10:2724. doi: 10.1038/s41467-019-10655-6 (PMC6586857; doi:10.1038/s41467-019-10655-6)
Supplement: Supplementary file 2 — Peer Review File [file 41467_2019_10655_MOESM2_ESM.pdf]

Editorial Note: This manuscript has been previously reviewed at another journal that is not operating a transparent peer review scheme. This document only contains reviewer comments and rebuttal letters for versions considered at Nature Communications.
